# DSG3 Facilitates Cancer Cell Growth and Invasion through the DSG3-Plakoglobin-TCF/LEF-Myc/Cyclin D1/MMP Signaling Pathway

**DOI:** 10.1371/journal.pone.0064088

**Published:** 2013-05-30

**Authors:** Yin-Ju Chen, Li-Yu Lee, Yin-Ka Chao, Joseph T. Chang, Ya-Ching Lu, Hsiao-Fang Li, Ching-Chi Chiu, Yi-Chen Li, Yan-Liang Li, Jeng-Fong Chiou, Ann-Joy Cheng

**Affiliations:** 1 Department of Radiation Oncology, Taipei Medical University Hospital, Taipei, Taiwan; 2 Translational Research Laboratory, Taipei Medical University Hospital, Taipei, Taiwan; 3 Department of Medical Biotechnology, Chang Gung University, Taoyuan, Taiwan; 4 Department of Pathology, Chang Gung Memorial Hospital, Taoyuan, Taiwan; 5 Department of Thoracic Surgery, Chang Gung Memorial Hospital, Taoyuan, Taiwan; 6 Department of Radiation Oncology, Chang Gung Memorial Hospital, Taoyuan, Taiwan; 7 Department of Radiology, School of Medicine, College of Medicine, Taipei Medical University, Taipei, Taiwan; 8 Cancer Center, Taipei Medical University Hospital, Taipei, Taiwan; Cincinnati Children's Hospital Medical Center, United States of America

## Abstract

Desmoglein 3 (DSG3) is a component of the desmosome, which confers strong cell-cell adhesion. Previously, an oncogenic function of DSG3 has been found in head neck cancer (HNC). Here, we investigated how this molecule contributes to the malignant phenotype. Because DSG3 is associated with plakoglobin, we examined whether these phenotypic alterations were mediated through the plakoglobin molecule. Immunoprecipitation and immunofluorescence staining revealed that DSG3 silencing disrupted its interaction with plakoglobin and induced plakoglobin translocation from the cytoplasm to the nucleus. Knockdown of DSG3 significantly increased the interaction of plakoglobin with the transcriptional factor TCF and suppressed the TCF/LEF transcriptional activity. These effects further conferred to reduced expression of the TCF/LEF downstream target genes, including c-myc, cyclin D1, and MMP-7. Functional analyses showed that DSG3 silencing reduced cell growth and arrested cells at G0/G1 phase. Besides, cell migration and invasion abilities were also decreased. These cellular results were confirmed using tumor xenografts in mice, as DSG3 silencing led to the suppressed tumor growth, plakoglobin translocation and reduced expression of TCF/LEF target genes in tumors. Therefore, our study shows that the desmosomal protein DSG3 additionally functions to regulate malignant phenotypes via nuclear signaling. In conclusion, we found that DSG3 functions as an oncogene and facilitates cancer growth and invasion in HNC cells through the DSG3-plakoglobin-TCF/LEF pathway.

## Introduction

Desmoglein 3 (DSG3) is one of the components of the desmosome. Desmosomes are button-like points of intercellular contact that allow the attachment of cytoskeletal elements to the plasma membrane at sites of cell-cell. By anchoring to stress-bearing intermediate filaments, desmosomes provide strong intercellular adhesion to maintain tissue integrity and homeostasis [1]–[3]. Desmosomes are composed of proteins from at least three distinct gene families: cadherins (e.g., DSG1-4 and DSC1-3), armadillo proteins (e.g., plakoglobin and various plakophilins), and plakins (e.g., desmoplakins, envoplakin, and periplakin). These desmosomal proteins are coordinated and associated with one another to form the desmosome. The resulting supracellular scaffolding plays a key role in providing mechanical integrity to tissues [1]–[3]. In addition to their role in cell-cell adhesion, the cadherin and armadillo proteins may function as molecular transducers to convert an extracellular event into intracellular signals [4]. For example, the tail of DSG3 has been shown bound plakoglobin [1]–[3]. Plakoglobin is closely related to β-catenin, which is a well-known downstream effector molecule in the canonical Wnt signaling pathway [5]. Therefore, it is possible that DSG3 may transduce molecular messages through the plakoglobin signaling pathway.

Several reports have found that desmosomal proteins are abnormally expressed in various cancers. While some investigators have reported that the expression of desmosomal proteins is decreased in cancers, others have found that the expression is increased. For example, it has been reported down-regulated of DSC2 in colorectal cancer [6], DSC3 in breast and oral cavity cancer [7], [8], and DSG2 in gastric cancer [9], [10]. However, over-expression of DSG2 or DSG3 has also been shown in several cancers including skin, prostate, lung and head-neck cancer [11]–[14]. All these studies indicate that the dysregulation of desmosomal proteins plays a role during carcinogenesis. Consistent with other reports, we have previously found that DSG3 functions as an oncogene in head and neck cancer and is associated with advanced clinical stage [15]. In this study, we further investigated how this molecule contributes to cancer formation. Our results showed that DSG3 promotes cancer cell growth and invasion through a plakoglobin-mediated signaling pathway. These effects resulted in alteration of the TCF/LEF transcriptional activity and thus altered the expressions of downstream molecules, including c-myc, cyclin D1, and MMP-7, which may lead to malignant phenotypes.

## Materials and Methods

### Cell lines, shRNA construction, and cellular transfection

Two head and neck cancer cell lines, OECM1 and SAS [16], were used. The OECM1 cells were maintained in RPMI 1640 media, and the SAS cells were cultured in Dulbecco's Modified Eagle's Media. All media was supplemented with 10% fetal bovine serum (FBS) and antibiotics (100 U/ml penicillin, 100 U/ml streptomycin, and 0.25 g/ml amphotericin B), and cell lines were cultured in a humidified atmosphere at 37°C with 5% CO_2_.

The shRNA sequence targeting DSG3 (shDSG3), which has been previously described [15], was subcloned into a pCI-neo plasmid and used to establish the shDSG3 stably transfected cells. Plakoglobin targeted shRNA was designed as a 22-nt sense and antisense hairpin that was complementary to the plakoglobin mRNA sequence 5′-GGA TGC CCA GCG CCA CGT AGC T-3′ and was cloned into the pTOPO-U6 plasmid vector, as previously described [15].

For the plasmid transfection, cells were seeded at a density of 5×10^5^ in a 100 mm dish and cultured for 16 hours. When the cells reached 60% confluency, they were transfected with 6 µg of shRNA plasmid or the empty vector plasmid using Lipofectamine 2000 (Invitrogen, Carlsbad, CA) in Opti-MEM reduced serum media (Invitrogen, Carlsbad, CA). After 16 hours, the Opti-MEM media was replaced with fresh complete media. The stable transfected cellular clones were selected using a neomycin reagent, G418 antibiotic solution (Sigma, St Louis, MO, USA).

### Patients and determination of protein expressions in clinical tissues

The study was approved by the Institutional Review Board of Chang Gung Memorial Hospital, and written informed consent was obtained from all participants. Nine biopsy tissues from head-neck cancer patients visited at the Head-Neck Surgery clinics at Chang Gung Memorial Hospital (Taoyuan, Taiwan) were obtained, including four grossly normal mucosa and five cancer samples. Tissue proteins were extracted and subjected to immunoblot analysis for the determination of DSG3, plakoglobin, c-myc, cyclin D1, and MMP-7 as described below.

### Cellular protein extraction, immunoprecipitation and immunoblot analysis

The methods of protein extraction, immunoprecipitation and immunoblot were performed similarly as previously described [17], [18]. Briefly, cells were homogenized in CHAPS lysis buffer, incubated on ice for 30 minutes, and centrifuged to obtain the cellular proteins. For fractionation of nuclear proteins, the commencial NE-PER nuclear and cytoplasmic extraction reagents (Pierce Biotechnology, Rockford, IL) were used, according to the manufacturer's suggested protocol. To prepare the immunoprecipitation beads, 30 µl of protein A/protein G sepharose beads were conjugated with 4 µg of specific antibodies (clone 5H10 for DSG3, clone H80 for plakoglobin, clone H125 for TCF4, Santa Cruz Biotech, CA). For immunoprecipitation, 1 mg of the cellular protein extract was incubated with 30 µl of the specific protein A/protein G beads for 4 hours at 4°C. The beads were collected by centrifugation, resuspended in a sample buffer, and subjected to immunoblot analysis. Non-immunized mouse or rabbit serum IgG were used as a negative control to determine the specific effect of immunoprecipitation.

For immunoblot analysis, 30 µg of cellular protein was separated using 8% SDS-polyacrylamide gel electrophoresis and transferred to a nitrocellulose membrane. The membrane was hybridized with one of the following primary antibodies: clone 5H10 for DSG3, clone H1 for plakoglobin, clone M-20 for Cyclin D1, clone 9E10 for c-myc (Santa Cruz Biotech, CA), or clone MAB3315 for MMP-7 (Millipore, MA). The membrane was subsequently incubated with a secondary antibody conjugated with horseradish peroxidase (Santa Cruz Biotech, CA). The protein image was developed using a Renaissance Western Blot Chemiluminescence Reagent Kit (NEN Life Science Products, MA) and autoradiography. The density of each protein band was determined after normalization to an Actin control band by using Gel Image System and Image J software (Scion Corporation, MD).

### Cell growth, colony formation, and flow cytometry analysis

The growth and colony formation assays were performed as previously described [19], [20] Cells were seeded at a density of 5×10^5^ in 100 mm dishes, and the extent of cell growth was determined daily using a hemocytometer. For the colony formation analysis, a total of 1000 cells were seeded in 6-well plates and allowed to grow without being moved for 7 days in complete culture media containing 20% FBS. The number of cell colonies was counted after staining with 5% crystal violet for 15 min.

Flow cytometry analysis was performed as previously described [21]. Briefly, after synchronization of cells at the G0/G1 phase by serum starvation, cells were subsequently harvested at different time points (a 6-hour interval for OECM1 cells and a 3-hour interval for SAS cells). The cell pellets were fixed with ethanol, permeabilized with Triton X-100 and RNase, and then incubated with propidium iodide (PI) for nuclear staining. Samples were immediately analyzed using a FACScan flow cytometer (Becton Dickinson, San Jose, CA). The distribution of the cell cycle phases was determined using the Cell Quest Pro and the ModiFit software. Two independent experiments were performed for each data set.

### Cell migration and invasion assays

The cell migration and invasion assays were performed as previously described [19]. Briefly, cell migration assays were performed using Transwell polycarbonate chambers (Becton Dickinson Biosciences). Cells were seeded in a 6-well plate with Transwell inserts that had a porous polycarbonate membrane (8 µm size) and incubated in regular media. After 24 hours, cells that had migrated to the underside of the Transwell inserts were fixed with glutaraldehyde, stained with crystal violet, and photographed.

The cell invasion assay was performed using the BD BioCoat Matrigel invasion chambers (Becton Dickinson Biosciences, Bedford, MA) and the Millicell invasion chamber (Millipore Corporation, Bedford, MA). The membrane of the Millicell upper chamber insert, which has a pore size of 8 µm, was placed in a 24-well plate and coated with Matrigel. Cells were seeded in the upper chambers with media containing 1% FBS. The lower chambers contained complete culture media (10% FBS) to trap the invading cells. The invasion ability of cells was determined every 24 hours by counting cells in the lower chambers that had successfully passed through the Matrigel-coated membrane.

### Immunofluorescence and confocal microscopy

The immunofluorescence and confocal microscopy analysis was performed as previously described [22]. Briefly, the glass coverslips were first coated with poly-L-lysine and formaldehyde. The fixed cells were subsequently permeabilized with Triton-X-100 and blocked with fetal bovine serum. The coverslips were incubated with an anti-DSG3 (clone 5H10), an anti-plakoglobin (clone H-80, Santa Cruz Biotech), an anti-β-catenin (clone E5), or an anti-TCF4 (cloneD4) antibody, and stained with fluorescein isothiocyanate (FITC)- or a rhodamine-conjugated secondary antibody. The coverslips were mounted with mounting media containing DAPI (Vector Laboratories, Burlingame, CA). The fluorescence was visualized using a confocal laser microscope (Leica TCS Sp2 MP).

### Luciferase reporter assay for TCF promoter activity

To determine the TCF promoter activity, the TOPflash (TOP) and the negative control counterpart FOPflash (FOP) reporter plasmids (Millipore, Corporation, Bedford, MA) were used. The TOP plasmid contained TCF binding sites, while the FOP contained mutant, inactive TCF binding sites. The shDSG3 stable cells or plakoglobin knockdown cells were transfected with TOP or FOP reporter plasmids, along with the thymidine kinase promoter-Renilla luciferase reporter plasmid (Promega, Madison, WI) as an internal control. After 48 hours, the cells were harvested, and the TOP/FOP activities were measured using the Dual-Luciferase Reporter Assay System in combination with the GloMax 20/20 Luminometer according to the manufacturer's instructions (Promega). The TOPflash or FOPflash activity was normalized against the Renilla luciferase activity, and the fold increase in the TOP compared to the FOP reporter plasmid (TOP/FOP) was reported.

### Mouse xenograft models and immunohistochemical analysis of xenografted tumors

All animal procedures were approved by the IACUC (Institutional Animal Care and Use Committee) at Chang Gung University and followed the guidelines of our institution's research council for the care and use of laboratory animals. A minimal number of mice were used for the study and all efforts were made to minimize suffering. The method of generating xenografts was performed as previously described [23]. Briefly, a total of 6 male BALB/C null mice at 5 weeks of age were used for the experiment. A total of 5×10^5^ stable DSG3 silencing (shDSG3) cells or stable vector transfected cells were subcutaneously injected into the upper portion of the hind limb. The tumor size was monitored daily by calculating the volume as length×width×height using calipers. The tumor weight was measured after the mice were euthanized on day 38. The xenograft tumors were removed and subjected to immunohistochemistry (IHC) or western blot analysis.

IHC was performed as previously described [24]. Briefly, tissue samples were fixed in a formaldehyde solution and embedded in paraffin. The sections were deparaffinized with xylene, boiled in citrate buffer to retrieve the antigens, and blocked with hydrogen peroxide. Slides were then incubated with primary antibodies. The following primary antibodies were used: anti-DSG3 (clone 32-6300, Zymed Laboratories Inc., CA, USA), anti-c-myc (clone 9E10, Santa Cruz Biotech, CA, USA), anti-cyclin D1 (clone M20, Santa Cruz Biotech, CA, USA), or anti-MMP-7 (MAB3315, Millipore, Corporation, Bedford, MA). IHC analysis and color development were performed using a Dako Envision system (Dako, Carpinteria, CA) following the manufacturer's instructions. Antibody diluents were substituted for the primary antibodies as negative controls and then counterstained with hematoxylin (HE). The staining reactions were determined by microscopic examination.

### Statistical analysis

The statistical analyses were conducted using Student's t test to compare two sets of data between different samples. All p values were two-sided. A *p* value<0.05 was considered statistically significant.

## Results

### DSG3 silencing disrupts its interaction with plakoglobin and induces plakoglobin translocation to nucleus

**Figure 1:**
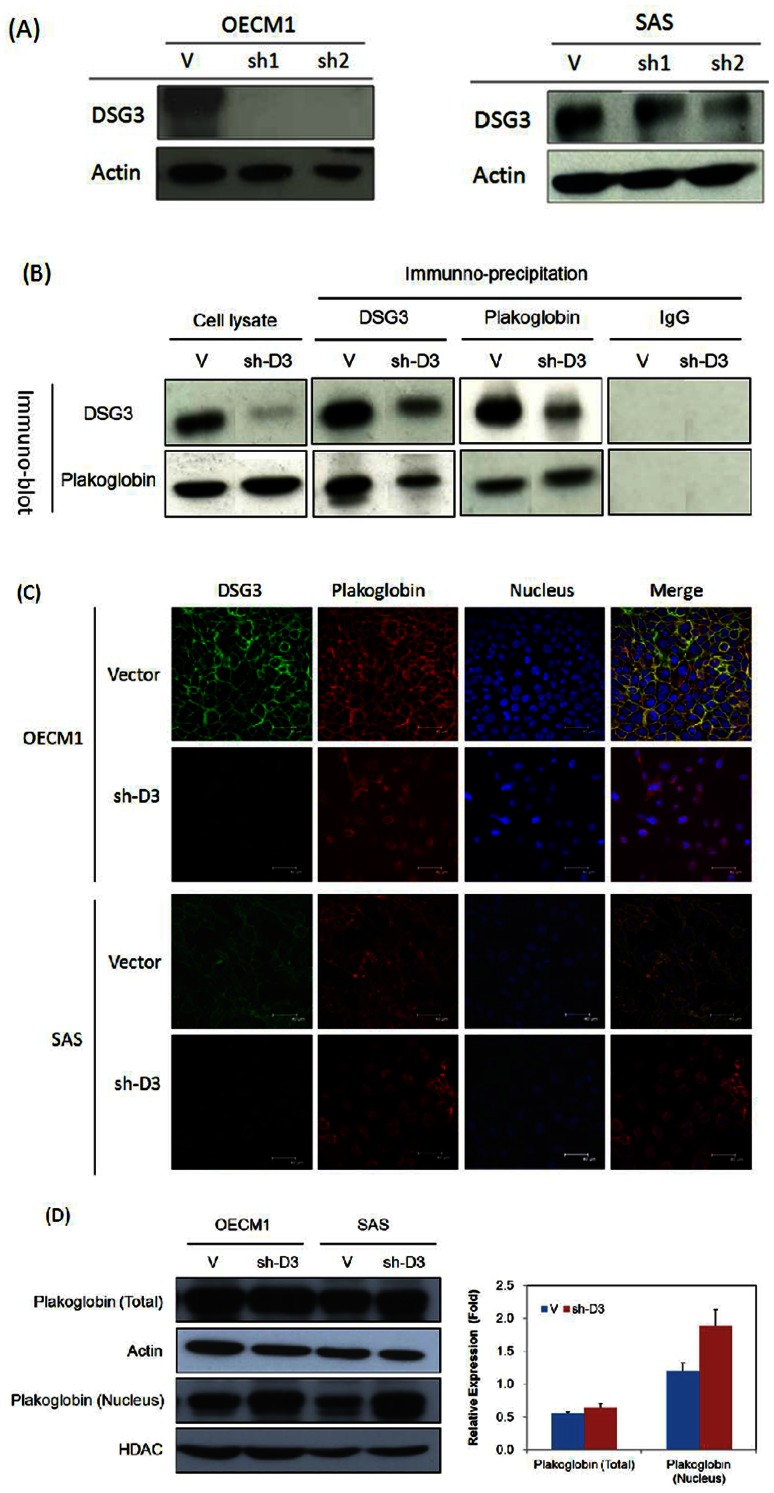
DSG3 silencing disrupts the interaction of DSG3 with plakoglobin and induces plakoglobin nuclear translocation. (**A**) DSG3 expression was suppressed in shDSG3 cells using RNAi. OECM1 and SAS cells were transfected with the DSG3-specific shRNA expression plasmid or the empty vector plasmid, and clones were selected using G418. Four clones (OECM1-sh1 and OECM1-sh2 in OECM1 cells and SAS-sh1 and SAS-sh2 in SAS cells) of shDSG3 cells were chosen and analyzed using western blot assays to determine the DSG3 protein level. Two clones (OECM1-V in OECM1 cells and SAS-V in SAS cells) of the vector-transfected cells were also chosen as controls. The actin protein level was determined as an internal control for protein expression. (**B**) DSG3 silencing reduced the interaction of DSG3 with plakoglobin, as determined by immunoprecipitation (IP) and immunoblot (IB) analysis. Two sets of cells were examined, empty vector-transfected cells and shDSG3-transfected cells. In each sample, proteins were extracted and immunoprecipitated with anti-DSG3 (D3), anti-plakoglobin (Pg), mouse IgG (IgG) (as a negative control) as indicated above each lane. Each sample was subsequently immunoblotted with either a plakoglobin (Pg)- or DSG3 (D3)-specific antibody, as indicated on the left side of each set of samples. (**C**) DSG3 silencing induced plakoglobinn translocation from the cytoplasm to the nucleus. Immunofluorescence and confocal microscopy were used to examine the localization of DSG3 and plakoglobin in both empty vector-transfected and shDSG3-transfected cells. DSG3 was detected using a DSG3-specific primary antibody with a FITC-conjugated secondary antibody and is shown in green. Plakoglobin was detected using a plakoglobin-specific primary antibody with a rhodamine-conjugated secondary antibody and is shown in red. DAPI staining was performed for nuclear staining and is shown in blue. The images were merged to identify the subcellular localization of DSG3 and plakoglobin. The scale bar indicates the size as 40 µm. (D) Total and nuclear protein extract were used to examine plakoglobin level by immunoblot assay. Protein density was measured by Image J software. Total protein extracts were normalized with actin; nuclear protein extracts were normalized with HDAC to calculate relative expression level.

To demonstrate the cellular function of DSG3, we established shDSG3 stably expressing cells derived from selection of the HNC cell lines OECM1 and SAS after transfection of shRNA targeting DSG3. The efficacy of DSG3 knockdown is shown in Figure 1A. In OECM1 cells, both clones (sh1 and sh2) displayed significant knockdown of DSG3 (>90%) compared to the cells transfected with the empty vector. In the SAS cells, sh1 and sh2 reduced DSG3 expression to 60% and 30%, respectively. Therefore we used OECM1-sh1 and SAS-sh2 clone cells throughout further study.

Since DSG3 is a component of the desmosome and interacts with other desmosomal proteins, such as plakoglobin, to maintain cell-cell adhesion. We investigated whether the signaling pathway of DSG3 were mediated through its interaction with the downstream molecule plakoglobin. As shown in Figure 1B, DSG3 silencing had no significant effect on the expression level of plakoglobin. When protein samples from the empty vector-transfected cells were immunoprecipitated with a DSG3-specific antibody and immunoblotted with a plakoglobin-specific antibody, these two molecules interacted with one another. In the shDSG3 cells, DSG3 exhibited a much weaker association with plakoglobin than in the empty vector-transfected cells. Similar results were also found in the reverse experiment. When protein samples were immunoprecipitated with a plakoglobin-specific antibody and immunoblotted with a DSG3-specific antibody, although interactions were found between these two molecules in the empty vector-transfected and the shDSG3 cells, the shDSG3 cells showed a much weaker association. These results indicated that DSG3 knockdown disrupts the interaction between DSG3 and plakoglobin.

Disruption of the interaction between DSG3 and plakoglobin in sh-D3 cells was further demonstrated using immunofluorescence. As shown in the merged nuclear staining and fluorescence images in Figure 1C, DSG3 and plakoglobin co-localized at the cell membrane in the control cells. In the sh-D3 cells, this co-localization was not observed. Instead, knockdown of DSG3 facilitated plakoglobin translocation from the cell membrane of desmosome junctions to the nucleus. Furthermore, total protein and nuclear protein extracts were examined plakoglobin distribution by immunoblot assay. In Figure 1D, plakoglobin level elevated in nuclear protein extract in sh-D3 cells than vector control cells, but the amount of plakoglobin in total protein fraction found no significant difference between sh-D3 and vector control cells. These results suggested that DSG3 knockdown disrupts its interaction with plakoglobin and induces plakoglobin translocation to the nucleus

### DSG3 knockdown increases the interaction of plakoglobin with the transcriptional factor TCF

**Figure 2:**
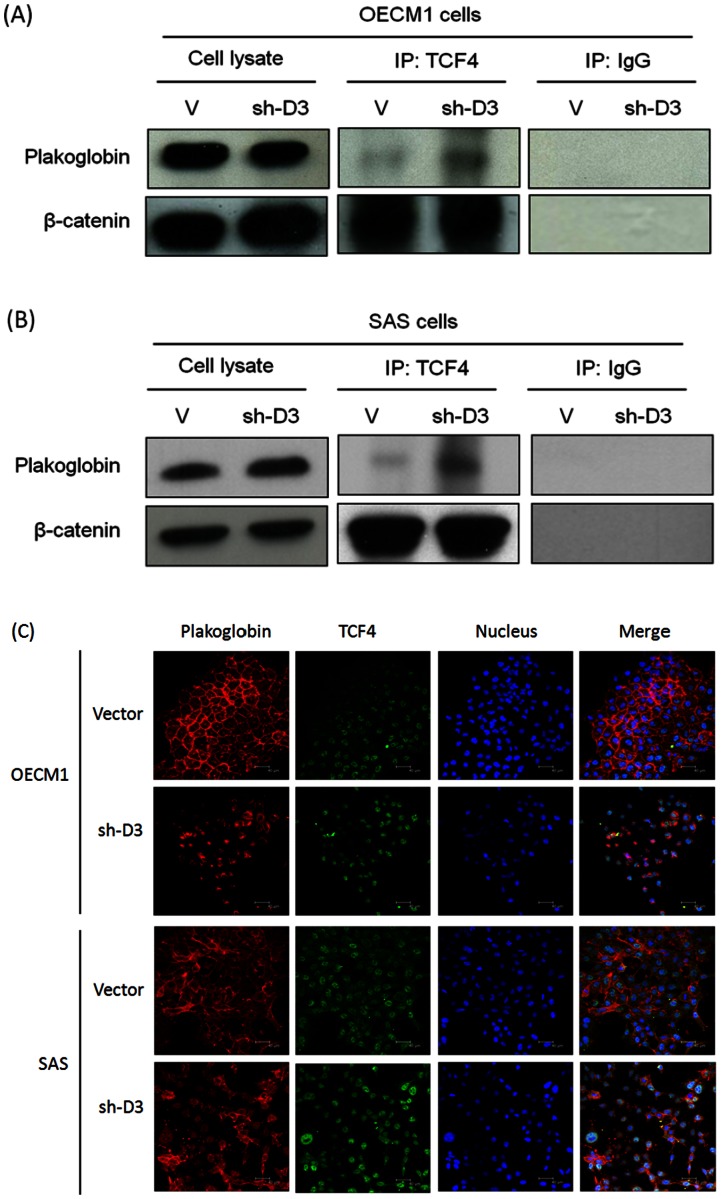
DSG3 silencing increased the interaction of plakoglobin with the transcriptional factor TCF. (**A, B**) Knockdown of DSG3 increased plakoglobin binding to the TCF4 transcriptional factor, as determined by immunoprecipitation (IP) and immunoblot (IB) analysis as determined in OECM1 (A) and SAS (B) cells. Cellular extracts of the vector or the shDSG3 stable transfected cells were immunoprecipitated with anti-TCF4, rabbit IgG (IgG) (as a negative control) and subsequently immunoblotted with plakoglobin or β-catenin antibody. (**C**) Immunofluorescence and confocal microscopy were used to examine the interactions among plakpglobin and TCF4. The vector or shDGS3 stable transfected cells were co-stained with either plakoglobin and TCF4 antibodies. After washing, the slides were incubated with rhodamine- or FITC- conjugated second antibody. DAPI staining was performed for nuclear staining. The scale bar indicates the size as 40 µm.

Plakoglobin is the nearest vertebrate relative of β-catenin, which is a downstream effector molecule in the canonical Wnt signaling pathway, although they have different transactivation potentials [25]–[28]. Wnt signaling initiates a cascade of events that allows β-catenin to escape from proteasome-mediated degradation, which normally ensures that there is only a low basal level of β-catenin in the cytoplasm. β-catenin is then able to translocate to the nucleus where it complexes with the T-cell factor/lymphoid enhancer-binding factor (TCF/LEF) family transcription factors and activates transcription of target genes [25], [28]. We therefore investigated whether plakoglobin nuclear translocation triggered by knockdown of DSG3 effects the interaction between plakoglobin and the transcriptional factor TCF. The immunoprecipitation and immunoblot methods were used to examine in both OECM1 (Figure 2A) and SAS cells (Figure 2B). As shown, DSG3 silencing increased the binding of TCF4 with plakoglobin but it had no significant effect on the interaction with β-catenin. Immunofluorescence assays also revealed consistent results. Compared to the control cells transfected with vector, knockdown DSG3 increased the amount of plakoglobin in the nucleus, which had been shown prominent co-localization with transcriptional factor TCF4 (Figure 2C). These results suggested that knockdown of DSG3 induced plakoglobin translocation into nucleus, which led to increasing binding to the transcriptional factor TCF.

### DSG3 silencing suppresses TCF/LEF transcriptional activity and the downstream target genes c-myc, cyclin D1, and MMP-7

**Figure 3:**
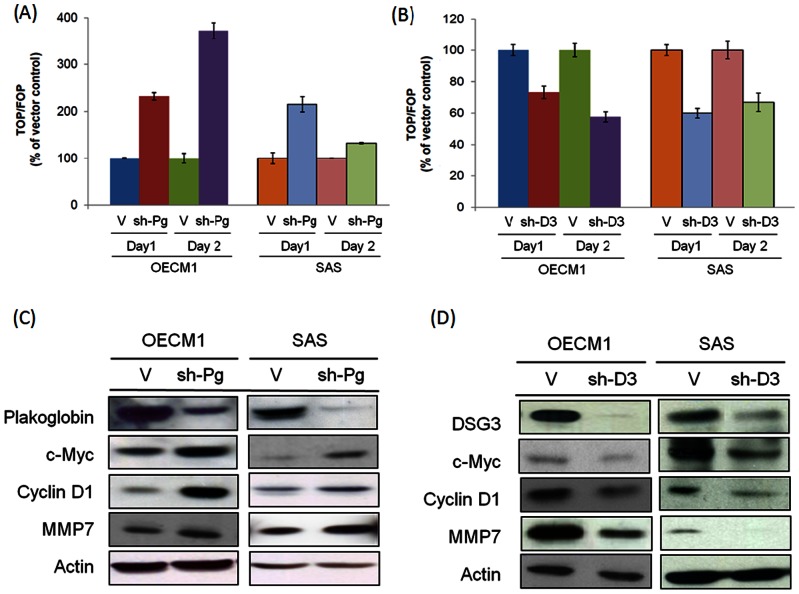
DSG3 silencing suppresses the TCF/LEF transcriptional activity and the downstream target genes c-myc, cyclin D1, and MMP-7. (**A**) and (**B**) The effects of TCF/LEF transcriptional activity after Plakoglobin or DSG3 knockdown. The stable plakoglobin silencing (sh-Pg) or DSG3 silencing (sh-D3) cells were transfected with TOPflash or FOPflash luciferase reporter plasmid and the Renilla plasmid. After 24 hours, the luciferase activity was determined using the Steady-Glo Luciferase Reagent. The firefly luciferase activity was normalized against the Renilla luciferase activity and the fold increase of the TOPflash activity compared to the FOPflash activity was reported. (**C**) and (**D**) The expressions of the TCF/LEF downstream target genes c-myc and cyclin D1 was determined in the cells stably transfected with specific shRNAs target to plakoglobin (sh-Pg) or DSG3 (sh-D3) cells, compared to the vector transfected stably cells. In each sample, proteins were extracted and analyzed by western blot assays to determine the expression levels of c-myc, cyclin D1, and MMP-7.

We further investigated whether plakoglobin nuclear translocation triggered by DSG3 silencing also induced effects on the TCF/LEF transcriptional activity. Since the role of plakoglobin on the Wnt pathway is not well defined, we therefore identified the function of plakoglobin on the TCF/LEF transcriptional activity. The TOPflash/FOPflash luciferase reporter assay was used [28]. As shown in Figure 3A, knockdown of plakoglobin expression increased the TCF/LEF transcriptional activity over 2-fold in both OECM1 and SAS cells after 1 day. These results suggested that in contrast to β-catenin, plakoglobin functions as a negative regulator of Wnt signaling and the TCF/LEF transcriptional pathway.

The potential effect of DSG3 silencing on the TCF/LEF transcriptional activity was examined. As shown in the Figure 3B, knockdown of DSG3 suppressed the TCF/LEF transcriptional activity to a level of 53% of the control cells in OECM1 cells and 59% in SAS cells after 1 day. Together with previous studies, these results demonstrated that DSG3 knockdown disrupted its interaction with plakoglobin (Figure 1B), causing plakoglobin's nuclear import (Figure 1C), facilitating its interaction with TCF (Figure 2), and leading to the enhancement of its suppressive effect on the TCF/LEF transcriptional activity (Figure 3B).

To further investigate how DSG3 regulates the plakoglobin–TCF/LEF signaling pathway leading to alterations in cellular homeostasis, we examined several TCF downstream target molecules, including c-myc, cyclin D1, and matrix metalloproteinase 7 (MMP-7) [29]–[31], all of which possess important functions on cell growth and invasion. As determined by western blot analysis, plakoglobin silencing substantially increased the expressions of c-myc, cyclin D1, and MMP-7 in the OECM1 and SAS cell lines (Figure 3C), whereas DSG3 silencing suppressed the expressions of these proteins in both cell lines (Figure 3D). These results suggested that DSG3 silencing suppressed the TCF/LEF transcriptional activity, which would further inhibit the expressions of the downstream molecules c-myc, cyclin D1, and MMP-7.

### DSG3 silencing inhibits cell growth and induces cell cycle arrest at the G0/G1 phase

**Figure 4:**
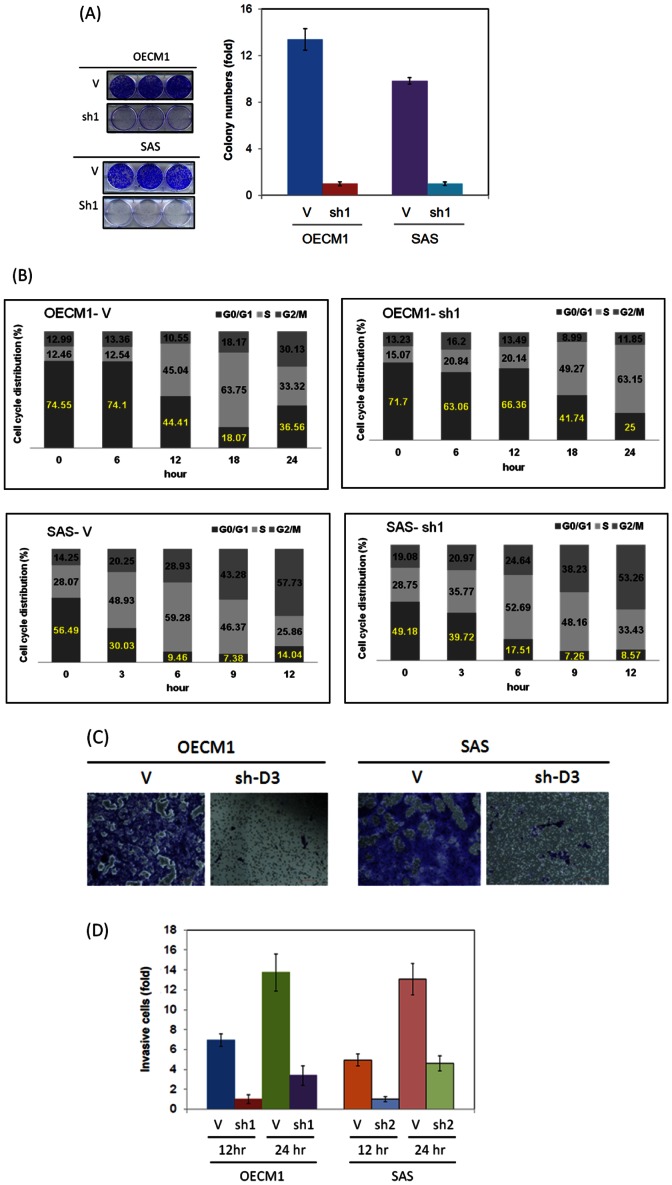
DSG3 knockdown inhibits malignant phenotypes. (**A**) Cell growth was reduced in shDSG3 cells. A total of 10^5^ cells of empty vector-transfected or shDSG3-transfected (sh-D3) cells were seeded into a 10 mm plate and cultured for 3 days. Experiments were performed in triplicate (***, p<0.001) (Upper panel). Colony formation was reduced in shDSG3 cells. A total of either 1000 empty vector-transfected cells or shDSG3-transfected (sh-D3) cells were seeded into a 6-well plate and incubated for 7 days to allow colony formation. Cell colonies were visualized and counted following staining with 5% crystal violet. (***, p<0.001) (Lower panel). (**B**) The cell cycle arrested at the G0/G1 phase in shDSG3 cells. The shDSG3 (sh-D3) cells (5×10^5^ cells per 10 cm dish) were synchronized in the G0/G1 phase by culturing them in serum-free media for 24 hours. The cell culture media was then changed to complete media to allow the cells to enter into the cell cycle. Cells were collected every 6 or 3 hours, and their cell cycle status was determined using flow cytometry. (**C**) The cell migration ability was reduced in shDSG3 cells, as determined using a Transwell migration assay. Either empty vector-transfected or shDSG3-transfected (sh-D3) cells were seeded at a density of 10^5^ cells per well in a 6-well plate with Transwell inserts that had a porous polycarbonate membrane (8 µm size) and incubated in regular media. After 24 hours, cells that had migrated to the underside of the Transwell inserts were stained with crystal violet. Each experiment was performed in triplicate. (***, p<0.001). (**D**) The cell invasion ability was reduced in shDSG3 cells, as determined using a Matrigel invasion assay. Either empty vector-transfected or shDSG3-transfected (sh-D3) cells were seeded at a density of 10^5^ cells per well in a 24-well plate with Matrigel-coated Millicell invasion chambers and incubated at 37°C for 24 hours. The numbers of cells that migrated through the Matrigel to the lower chamber were determined every 12 and 24 hours. Each experiment was performed in triplicate (***, p<0.001).

Since DSG3 has demonstrated as a molecular transducer to regulate the expressions of c-myc, cyclin D1 and MMP7, we examined whether this molecule functioned in cell growth regulation. As shown in the Figure 4A, DSG3 silencing suppressed the cell growth of OECM1 cells by 64% at day 3 and suppressed the cell growth of SAS cells by 76% at day 3. This phenomenon was further supported using colony formation assays that showed 90% growth inhibition in OECM1-shD3 cells and 80% growth inhibitions in SAS-shD3 cells. To investigate the effect of DSG3 on cell cycle regulation, flow cytometry analysis was performed. As shown in Figure 4B, knockdown of DSG3 resulted in cells that arrested at the G0/G1 phase, as illustrated by the delay in entering into S phase. OECM1 cells started entering into S phase at 12 hours (45.0%), whereas OECM1-shD3 cells did not start entering into S phase until approximately 18 hours (49.3%). Similarly, SAS cells starting entering into S phase at 3 hours (48.9%), whereas SAS-shDSG3 cells did not enter into S phase until approximately 6 hours (40.27%). These results suggested that DSG3 knockdown inhibited cell growth by arresting cells at the G0/G1 phase delayed enter to S phase.

### DSG3 silencing inhibits cell migration and invasion

Since DSG3 functions in cell-cell interactions, we examined whether DSG3 knockdown affected cell migration and invasion. The cell migration results from the Transwell assay are shown in Figure 4C. The migration of two sh-D3 cell lines was dramatically reduced after 24 hours when compared to the control cell lines. The cell invasion results from the Matrigel assay are shown in Figure 4D. The invasiveness of sh-D3 cells was also significantly reduced (86% in OECM and 71% in SAS cells after 12 hours). These results demonstrated that silencing of DSG3 significantly inhibited cell migration and invasion.

### DSG3 silencing suppresses the growth of xenografted tumors, which is associated with plakoglobin translocation and reduced expressions of TCF target genes

**Figure 5:**
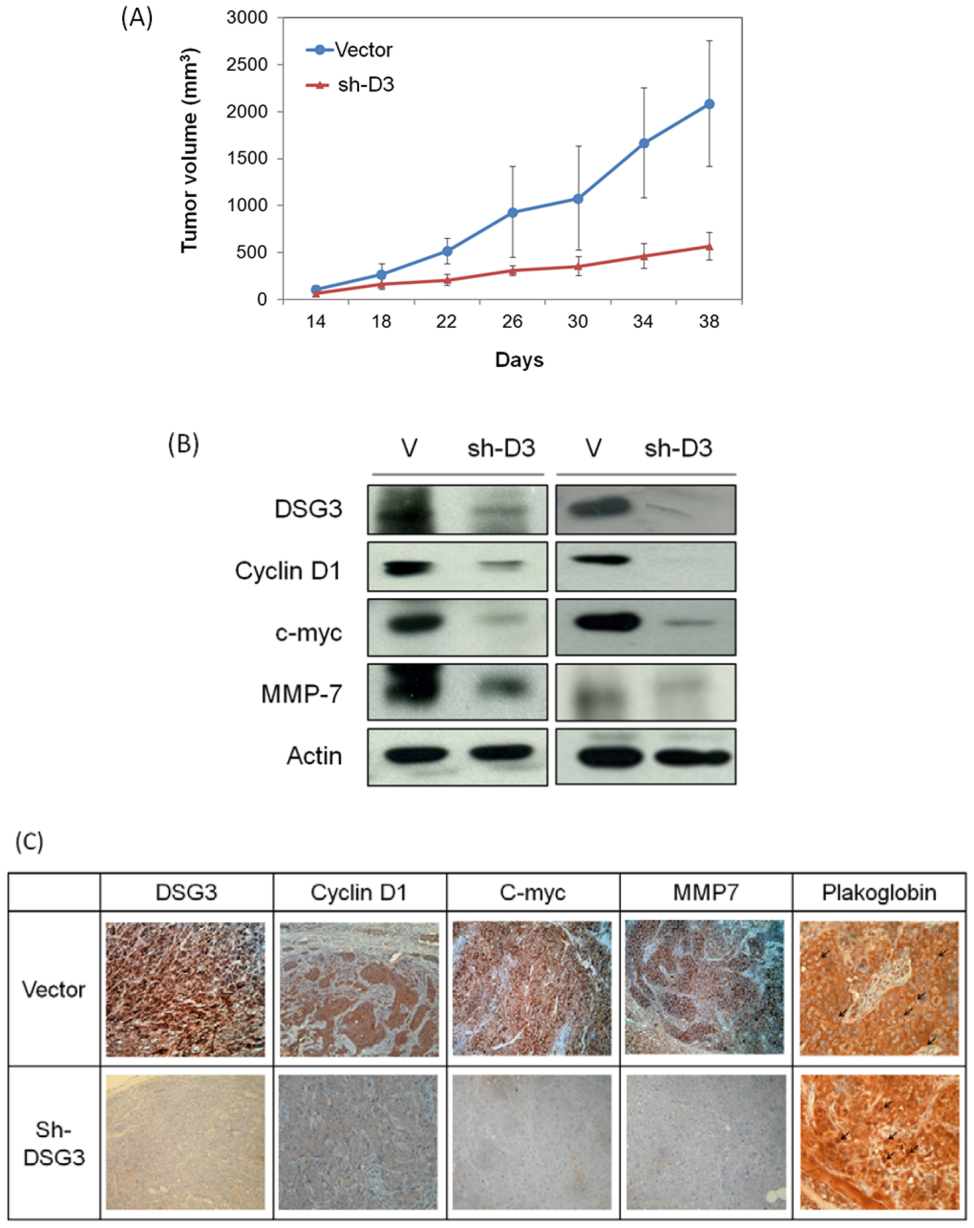
DSG3 silencing suppressed the growth of xenografted tumors, which is associated with plakoglobin translocation and reduced expressions of TCF target genes. (**A**) Six of each group BALB/C null mice were subcutaneously injected with 5×10^5^ cells of the SAS cell line stably transfected with the vector (V) or the shDSG3 (sh2) plasmids. The tumor size was measured every 4 days starting two weeks after injection. Each dimension of the tumors were measured by calipers, and the tumor size was calculated as length×width×height. (***: p<0.001). (**B**) Five weeks after tumor grafting, the tumors were removed subjected to western blot analysis for the expression of c-myc, cyclin D1, and MMP-7 proteins. (**C**) The grafted tumors were subjected to immunohistochemistry staining for the localization and expression of plakoglobin, c-myc, cyclin D1, and MMP-7 proteins.

To examine the effect of DSG3 silencing in vivo, we established xenografted tumors in BALB/C nude mice. The DSG3 knockdown stable cancer cell lines (SAS-sh2) and the empty vector transfection cell line as a control were subcutaneously injected into the mice. Tumor growth was assessed every 4 days. Figure 5A shows the average tumor volume over the 38 days of study between these groups. In the DSG3 silencing groups, tumor growth was sustained significantly slower compared to the controls. On average, SAS-sh2 decreased tumor growth by 50% at day 30 (*P*<0.001) and by 71% at day 38 (*P*<0.001). After mice being scarified, the tumor weight was measured as an average reduction of 63% in the DSG3 knockdown mice compared to control group (*P*<0.01).

To confirm that the suppression of tumor growth was associated with the knockdown of DSG3 due to plakoglobin translocation and the downstream molecules, the xenografted tumors were dissected and the protein expression was examined using western blot and immunohistochemistry assays. As shown in Figure 5B for two sets of xenografted tumors, the DSG3 protein was reduced in the shDSG3 xenografts compared to the higher levels in the vector-transfected xenografts. These results were accompanied with the decrease of the TCF target molecules c-myc, cyclin D1, and MMP-7. Figure 5C showed the results of immunohistochemistry staining of the dissected xenograft tumors. DSG3 silencing tumors exhibited a remarkable staining of plakoglobin in nucleus in contrast to the vector transfected tumors in the cell-cell boundary. Furthermore, this DSG3 silencing significantly suppressed the expressions of cyclin D1, c-myc, and MMP-7. These results demonstrated that DSG3 knockdown reduced cell growth in vivo through plakoglobin dependent inhibition of TCF/LEF transcriptional activity and downstream regulatory molecules.

### Elevation of DSG3 is associated with plakoglobin translocation in cancer tissues

**Figure 6:**
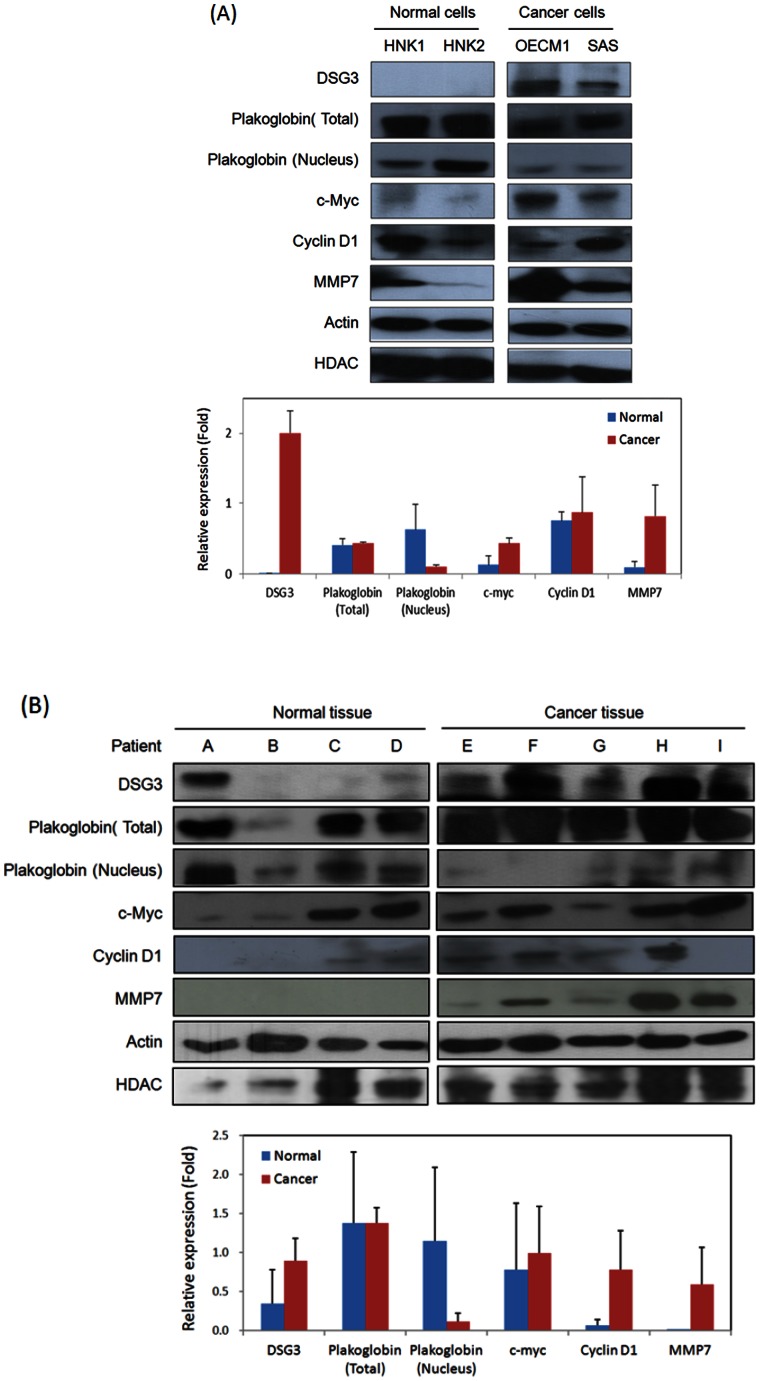
Elevation of DSG3 is associated with plakoglobin translocation in cancer tissues. (**A**) Cells from two clones of normal keratinocytes (HNK1, HNK2) and two cancer cell lines (OECM1, SAS) were examined. (B) Clinical tissue samples of four grossly normal biopsies and five pieces of cancer tissues from head-neck cancer patients were examined. Cellular or nuclear proteins were extracted and subjected to immunoblot analysis for the expression of DSG3, plakoglobin, c-myc, Cyclin D1 and MMP7, as described in the method section. Expressions of Actin served as internal control for cellular protein, and HDAC for nuclear protein. Cellular protein expression was normalized by Actin and nuclear protein by HDAC after determination of each band density by Image J software.

To determine whether DSG3 over-expression is related to plakoglobin nuclear translocation and the downstream protein expressions in clinical samples, we have analyzed the expressions of these molecules in several normal and cancer cell lines (Figure 6A) as well as in the grossly normal and cancer tissues from head-neck cancer patients (Figure 6B). Although differential levels of proteins between various samples, the cancer cells or tissues showed the same trend of expressions compared to the normal cells. In the cell line analyzed, DSG3 showed high expression in two cancer cell lines, in contrast to undetectable level in two normal keratinocytes. The relative expression of plakoglobin in nuclear fraction was decreased considerably in the cancer cell lines, but minimal alteration in the total protein extracts, suggestion that DSG3 over-expression was associated with plakoglobin translocation. The TCF/LEF downstream molecules c-myc, cyclin D1 and MMP7 were also increased significantly in cancer cells (Figure 6A). In the clinical tissues, four grossly normal mucosa and five cancer tissues were examined. Consistently, DSG3 over-expression and plakoglobin translocation was found in the cancer tissues, in concurrence with up-regulations of c-myc, cyclin D1 and MMP-7 (Figure 6B). In all, these results supported our molecular findings that DSG3 functions as an oncogene in head-neck cancer through the DSG3-plakoglobin-CF/LEF - myc/cyclinD3/MMP signaling pathway.

## Discussion

Desmosomes are intercellular junctions that offer strong cell-cell adhesion. However, the desmosomal proteins, such as DSG3, may also function as molecular transducers to convert extracellular signals. Clinically, the pathology underlying the autoimmune disease pemphigus vulgaris supports this concept. Autoantibodies against DSG3 have been found in skin keratinocytes from patients with pemphigus vulgaris. The binding of DSG3 with the autoantibodies disrupts the desmosomal structure, leading to the aberrant expression of plakoglobin and the overexpression of the downstream molecule c-myc [32]. Therefore, while desmosomal proteins are needed to maintain a physiological balance between cells, they also function as transducer molecules that can translate extracellular signals into cells. There is an supportive evidence showing that treatment of cell lines with a DSG3-specific antibody to neutralize the DSG3 signal results to increases of p38, MAPK, and PKC activations [33], [34]. Moreover, overexpression of DSG3 resulted in the formation of filopodial protrusions and increased cell migration [35]. In this present study, we found that DSG3 silencing significantly suppressed cell growth and mobility (Figures 4), suggesting that DSG3 plays oncogenic functions with multiple aspects. These results indicate that the abnormal expression of DSG3 protein during carcinogenesis may not only affect cellular adhesion but also affect signal transduction in complex signaling pathways.

The mechanism underlying these functions of DSG3 was further investigated. Since DSG3 and all other desmosomal proteins are closely associated with each other, we hypothesized that DSG3 silencing would disrupt the downstream desmosomal proteins, thereby leading to alterations of the signaling pathway. The armadillo family, such as plakoglobin and various plakophilins, has several associated proteins both within and outside of the desmosome. Because plakoglobin directly interacts with DSG3 [26], [27], we investigated whether the cellular effects of DSG3 were mediated through the plakoglobin signaling pathway. Dysregulations of plakoglobin have been previously reported in various cancers, although its function as a tumor suppressor or oncogene is paradoxical. The tumor suppressive functions, such as reduced expression and suppressed cell mobility, were found in breast, lung, and oral cancers [36]–[40]. However, over-expression and the ability to promote cell migration were reported associated with the aggressive types of hepatocellular and bladder cancers [41], [42]. In the present study, we found that DSG3 silencing reduced its interaction with plakoglobin (Figure 1B) and resulted in translocation of plakoglobin to the nucleus (Figure 1C). The nuclear translocation of plakoglobin, rather than the altered expression, plays an important role in further suppressing the oncogenic functions.

Plakoglobin, also named γ-catenin, is homologous with β-catenin, and both play a dual role in cell adhesion and signal transduction. In the Wnt signaling pathway, cytoplasmic β-catenin is phosphorylated by GSK3 and subjected to proteosomal degradation in the absence of Wnt signaling. Interaction of Wnt with its receptor blocks phosphorylation of β-catenin, allowing translocation to the nucleus, where it complexes with the TCF/LEF transcription factors and activates transcription of target genes to regulate cell fate decisions [25]. Accumulated evidences have demonstrated that activation of the Wnt/β-catenin signaling pathway promotes several types of cancer formation [43]. Although the function of β-catenin in mediating Wnt signaling is well established, the role of plakoglobin is still unclear. It has been previously reported that plakoglobin behaves as an oncogene in transformed rat kidney epithelial cells, probably due to the activation of the c-myc gene [44], in AML, plakoglobin through stabilization and nuclear localization of β-catenin regulate wnt signaling pathway [45]. However, this molecule has also been demonstrated to play the role of tumor suppressor by antagonizing the β-catenin induced transcriptional activity to inhibit tumorigenicity [39], [46], [47]. In agreement with later observations, we found that plakoglobin knockdown promoted the TCF/LEF transcriptional activity (Figure 3A), resulting in increased expression of downstream proteins, including c-myc, cyclin D1, and MMP-7 (Figure 3C). The reduction of plakoglobin level in nucleus was also found in the cancer cell lines and clinical tissues (Figure 6A–B). These results suggested that plakoglobin serves as a negative regulator of the Wnt signaling pathway in head and neck cancer cells.

**Figure 7:**
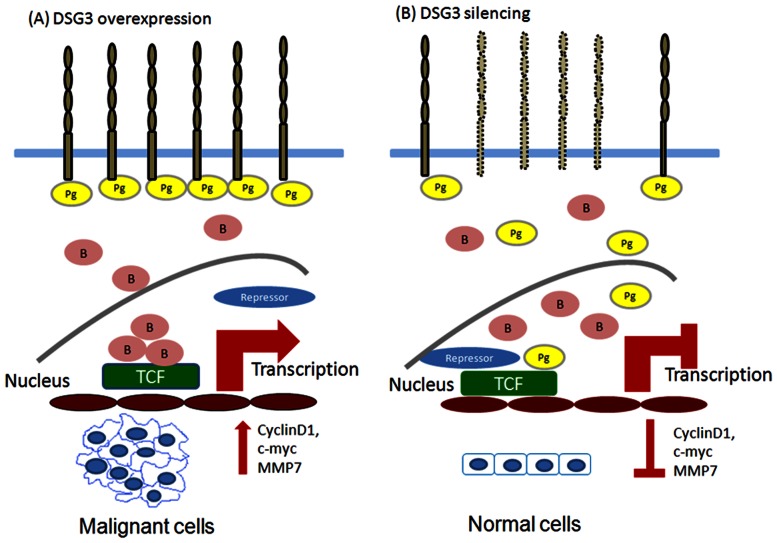
Hypothetical model illustrating the DSG3-plakoglobin signaling pathway modulating cell carcinogenesis in head and neck cancer. (A) In cancer cells, DSG3 is overexpressed and captures more molecules of plakoglobins in the membrane. Loss of the antagonist function of plakoglobin results in more molecules of β-catenin entering the nucleus, which subsequently activates the expression of TCF/LEF downstream target genes c-myc, cyclin D1, and MMP-7 resulting in malignant cells. (B) Silencing DSG3 by shRNA treatment reverses the effects of DSG3, resulting in translocation of plakoglobin to the nucleus thus rendering the negative regulatory effect of plakoglobin on TCF/LEF transcription. (Pg: plakoglobin; B: β-catenin).

Therefore, the role of DSG3 and the link from the desmosomal junction to the downstream signaling regulation was demonstrated in this study. We found that knockdown of DSG3 in cancer cells resulted in (1) disruption of its interaction with plakoglobin and induced plakoglobin translocation from the cell membrane to the nucleus (Figure 1), (2) an increase in the interaction of plakoglobin with the TCF transcriptional factor and suppressed the TCF/LEF transcriptional activity (Figures 2, 3), (3) inhibition of the expression of TCF/LEF downstream target genes c-myc, cyclin D1, and MMP-7 (Figures 3, 5), and (4) suppression of the malignant phenotypes, including cell proliferation, invasion, and tumor growth (Figures 4, 5). The inhibition of cyclin D1 may be associated with the suppression of cell growth and cell cycle arrest at the G0/G1 phase, while the inhibition of MMP-7 may lead to the effects on cell migration and invasion. These observations provide additional information in support of the concept that desmosomal molecule DSG3 exerts nuclear signaling molecules to regulate malignant phenotypes (Figure 7). In conclusion, the desmosomal protein DSG3 may function as a transducer molecule to translate extracellular signals into cells through plakoglobin-mediated signaling pathways. Over-expression of DSG3 in cancers may be just an initial step that affects the regulation of cell fate. DSG3 over-expression confers the recruitment of plakoglobin, resulting in the reduction of plakoglobin's tumor suppressive function (Figure 7A). Silencing DSG3 expression by shRNA can inhibit the actions of the downstream effector molecules and reverse the malignant phenotypes (Figure 7B). This study provides evidence that DSG3 could potentially serve as a molecular target for chemotherapeutic agents in the treatment of head and neck cancer.
